# Adulterated and Misbranded Foods*Read before the Detroit Academy of Medicine, February 26, 1918.

**Published:** 1918-08

**Authors:** Charles D. Aaron

**Affiliations:** Professor of Gastroenterology and Dietetics in the Detroit College of Medicine and Surgery, Detroit, Mich.


BUFFALO MEDICAL JOURNAL

Yearly Volume 74 AUGUST, 1918 Number 1

### ORIGINAL ARTICLES

**The right is reserved to decline papers not dealing with practical medical and surgical subjects, and such as might offend or fail to interest readers. Contributors are solely responsible for opinions, methods of expression and revision of proof.**

### Adulterated and Misbranded Food*

By CHARLES D. AARON, Sc. D., M. D., F. A. C. P.

Professor of Gastroenterology and Dietetics in the Detroit College of Medicine and Surgery, Detroit, Mich.

It is admitted by both manufacturers and consumers that artificial means are absolutely unnecessary for the preservation of food. But selfishness and politics have tried for years to discredit the good work done by the Bureau of Chemistry of the Department of Agriculture designed to promote the national health. The laity should know that the use of harmful drugs makes it possible for manufacturers to convert decaying fruits and vegetables into jellies and catsups which look good and taste fairly good, but are unwholesome. They should know that manufacturers take filthy, rancid, rotten, frowy butter and "renovate" it into so-called dairy and creamery butter.

The American Association for the Promotion of Purity in Food Products has declared that chemical preservatives are not necessary for the conservation of the essential features in food products.

That adulterated food does harm, no one can deny. Alum in bread, formaldehyde in milk, salicylic acid and benzoic acid in meats, borax in cheese, coloring matter in butter, acids in smoked meats, copper in pickles, etc., are simple illustrations. The medical press throughout the United States has commented on the subject—but widely varying opinions have been expressed as to the degree of harmfulness of the

*Read before the Detroit Academy of Medicine, February 26, 1918.

food preservatives. Some writers take the ground that boric and salicylic acid, even when contained in considerable quantities in foods, are much less harmful than the toxins of putrefaction which sometimes develop if the preservatives are not used. The French law on this subject regards the addition of any preservative to food as deleterious on the ground that any such substance must inhibit the action of the gastric and intestinal juices and thus delay digestion.

The subject, however, is a complicated one. One expert, for instance, declares that he believes the addition of large quantities of antiseptics to food is in the main deleterious, yet there are certain foods, he says, in which it is a distinct advantage. He cites catsup, which is ordinarily sold in a package of sufficient size to last a small family for several days, perhaps weeks. If no antiseptics were used in its preparation, as soon as the bottle was opened fermentation would be set up and the contents soon spoil. This condiment, he declares, is used in small quantities, and the amount of salicylic acid or other antiseptic any individual would obtain is inconsiderable. This reasonable statement of the value of antiseptics shows that no sweeping general rule can be applied in prohibiting the use of such substances. But their extensive use is admittedly harmful; in samples of butter examined, from sixty to eighty grains of boric acid to the pound have been found.

The necessity of preserving food-stuffs is more acute today than in early times, though there never was a time when it was not an urgent problem. Food is not always naturally available when needed. Not only must vegetables, fruits, and nuts be gathered, but their supply varies with the season of the year. Most foods require preparation to render them not only palatable, but even digestible; and evidently the aboriginal tribes in this latitude were under the necessity of storing summer food or doing without it at other seasons. But our difficulties increase as our necessities multiply. The great demand for "preserved" food in ancient times is evidenced by the fact that there were frequent famines; transportation was extremely slow and men were dependent upon the products of their husbandry.

Inasmuch as the succession of seasons, the intervals between harvests, and the distances from the sources of supply compel man to store and transport food products, and since these are more or less perishable, the necessity of artificially preserving these foods from decay is apparent.

Today ships must be provisioned, the camps, the army, navy and marine, the hospitals and places of detention; our allies must be supplied, and the preparation and preservation of food has developed on a large scale and into a specialty.

One of the most important and oldest foods is bread. When we get it from the baker's it has no very great keeping qualities, but in its original form it was one of the best preserved foods. The primitive bread was not fermented; it was simply the flour from some grain mixed with water and exposed to heat. Essentially we have the same thing today in the ordinary cracker, also in the "hard-tack" of the army and navy. In this particular civilization has made little progress.

There are two great reasons why food is adulterated: First, to give it an appeal to our fanciful taste; second, to make it more profitable to the manufacturer.

The adulteration of food for both these purposes has been known for generations, for we know that even the Romans adulterated food and traded it with the Greeks.

There are four methods of preserving meat:

1st. Curing with condimental substances.

2nd. Treatment with chemical substances.

3rd. Sterilization by heat.

4th. Drying.

Salt is the condiment most commonly used. Salt does not inhibit the enzymic action that tends to ripen meat. Sugar is occasionally used with salt.

2nd. Chemicals are used as germicides. Small quantities do not impart taste or odor to the meat; they make it look fresh on the outside while changes of a dangerous character may be going on within.

3rd. Sterilization by heat is the safest and best method.

4th. Drying is the most ancient and popular method.

The essential point in all these methods is that the food should from the start be free of all decomposition.

People do not seem to have the least conception as to the materials and methods formerly used in the adulteration of food. Sausages are adulterated to keep them from decomposing and at the same time give them that red color which is so attractive. "Freez-em" was a secret combination of salts sprinkled on roasts, chicken, geese, pork, ets., to keep them for a week. A solution of "Freez-em" will keep oysters fresh for a couple of weeks. "Cold Storine" and "Bull Meat Flour" are "guaranteed" meat preservatives. "Best Bull Beef Binder" makes two hundred pounds of sausage out of one hundred pounds of meat and preserves it under all conditions of the weather for ten days. The coloring used under various names is mainly carmin.

Much of the smoked meat sold has never seen the inside of a smoke-house. Some of the substances used to give it the smoky flavor are "Zanzibar Carbon," "Deems," "Mallory's," "Krauser's" and many others. To smoke the meat, simply hang it up and paint it every day for three days with one of the above-named solutions. At the end of this time the meat will be smoked most effectively—of a good color and an agreeable odor and taste. Pyroligneous acid, which is crude wood acetic acid, is the main ingredient in these preservatives. Sodium sulphite, calcium sulphite, boric and salicylic acids and benzoic acid with coloring matter are other preservatives. Clams, lobster, and fish can all be treated with these chemicals.

In all canned meat it is wise to test the reaction of the jelly. If this be alkaline then decomposition has taken place and the meat is unfit for use. Another test is to open the can under water; if any bubbles rise, this is a sign that the meat has undergone putrefaction and is unfit for use. One great danger of artificial preservatives is due to the fact that they do not penetrate to the bone. This part will decompose and toxins be produced. The natural method of preservation affects all the parts. The smallest particle of bacterial toxin is sufficient to produce prostration and poisonous symptoms of great severity.

Much of the so-called Russian caviar is made from sturgeon roe at Sandusky, Ohio, or at some port on the great lakes. In some cases the caviar is made in Russia from eggs sent there from America. Very little real Russian caviar finds its way into the American market.

Much of the Camembert cheese, supposed to be imported, is really made in the United States. The law now compels the manufacturer to state on the label where it came from. When this law went into effect the Camembert makers had a large stock of labels on their hands. Unblushingly they defaced the fine Parisian French on the labels and printed across it in very black ink the words "Camembert Type Cheese. Made in the United States." Now, however, they must state in good English, that the cheese is as near like that made in France as possible, and the consumer knows what he is getting.

Formaldehyde is the preservative usually added to milk. It evaporates when the containers are open. For this reason larger quantities of it are used in open containers than in closed ones. "Freez-em," "Busy Bee," "Giant" and many other so-called secret preparations owe their efficacy to formaldehyde. Salicylic acid and boric acid are quite commonly used as preservatives. Gelatin is the basis of substances used to adulterate cream to make it thicker. It is sold in the shops under the name "Eureka" or "Victor." It is also frequently used to thicken skim milk. Bismarck Brown is the coloring matter used for coloring butter, and there are many vegetable colorings used. Butterine and oleomargarine are artificial butter, made up of oleo (from beef fat) churned with lard, milk and salt. The oleo oil, however, frequently contains cotton-seed oil, so that even oleomargarine may be adulterated. Paraffin is frequently added to oleomargarine to give it a firm appearance. Renovated butter is old butter melted and treated with air to remove the rancid odor. It is frequently kept in the shops in small tubs, and sometimes sold as dairy butter. The paper wrapped around the tub, impressing the customer as an evidence of cleanliness, serves to cover the label.

In canned goods the object is to retain the taste and color of the original article. Bulging of a can is quite suggestive of decomposition. Catsup, sauces and pickles have coloring added to them. Alum is added to pickles to harden them. Horseradish and turnip are added to catsup as an adulterant. Copper sulphate is added to give the green color to pickles and the boiling of peas in a copper kettle will cause them to retain their green color.

Jams and jellies are adulterated with refuse apple jelly. Coloring matter and gelatin are added to make them attractive.

Honey has been palmed off on the public which was only 1% genuine—on top—the rest being glucose. This adulteration is a fraud on the public, but it is not injurious to health. Gelatin, cane sugar, and glucose are used to adulterate honey. Stained honey is easily adulterated. A simple test is to add strong alcohol; if the specimen is pure honey, it will mix with only a slight precipitate, while glucose leaves a heavy precipitate of dextrin.

Glucose and coloring matters are used in nearly all candies as adulterants. Syrups are adulterated with glucose. Cheap cane sugar is colored with caramel. Lard is adulterated with cotton-seed oil and beef fat. Tallows and fats bleached absolutely white mix easily with lard.

The above illustrations will give an idea of how food has been adulterated in this country. After many debates a pure food law was finally secured. Sentiment had at last been aroused and a bill was passed. We can hardly realize the tremendous opposition which financially interested parties put up against the bill. The manufacturers of food preparations soon found it more difficult to humbug the people. The pure food bill was passed by both houses of congress in 1906. It prevents the manufacture, sale or transportation of adulterated, misbranded, poisonous or deleterious foods, drugs, medicine, and liquors. Immediately after the passage of this law the United States Department of Agriculture began to regulate its operation by designating what, according to the meaning of the law, is adulterated and what is not. It was also necessary to decide what constituted misbranding. This was an enormous work and it has not yet been entirely completed. To give you some idea of the many discriminations that must be made, let me say that sugar can be used in the preparation of any food product, but not for fraudulent purposes. For example, the addition of sugar to sweet corn in canning is forbidden unless the fact is stated on the label. Vegetables kept green by the use of copper are allowed to enter the United States if the label states this fact. Flour blended by the use of nitrogen peroxide is adulterated and cannot be sold under the pure food law.

After a long series of experiments conducted by the government through one of its bureaus designed by law for the purpose, and following the enactment of the food and drugs act, articles of food preserved by benzoate of soda were excluded from interstate commerce as being deleterious to the public health.

This action on the part of the government was embarrassing to two distinct commercial interests—namely, the manufacturers of benzoate of soda and the manufacturers of food products of inferior quality. These interests protested to the government that the original experiments were either defective or at least not conclusive that benzoate of soda as a food preservative was deleterious within the meaning of the law, and demanded a re-investigation of the subject at the hands of an independent commission. The government acceded to this demand and appointed several of the best known physiologic chemists as a referee board of consulting scientific experts with instructions to determine whether or not a food to which benzoate of soda had been added in either large or small quantities contains, by virtue of that fact, "any added poisonous or other deleterious ingredients which may render said food injurious to health." This referee board then conducted, or caused to be conducted, a series of experiments and at the conclusion of these experiments filed a report unreservedly favorable to benzoate of soda as a food preservative. The government, being virtually precommitted to the findings of its own referees, was thereby induced to rescind its decision and on March 3, 1909, issued a decision to the effect that no objection would be raised to "the use in food of benzoate of soda provided that each container or package of such food is plainly labeled to show the presence and amount of benzoate of soda."

This decision is far-reaching. It affects not only many millions of capital, but the health and lives of many more millions of people. It involves, furthermore, so serious a step as the reversal by a great government of its previously deliberately determined policy. The government may go without criticism as being justified by the report of its chosen referees, but the report upon which so momentous a decision was based must become the object of critical scrutiny.

It is important to remember that benzoate of soda, by virtue of its property as a powerful antiseptic, was used and, since the removal of the ban by the government, is again being used as a preservative of food. Experience amply proves that sound fruits and vegetables, including catsup from sound tomatoes, can be made to keep without the addition of benzoate of soda or any other deleterious medicament. Unsound fruit and slush from canning factories, slush that ought to go only into the sewer, can now be preserved, sold, and extensively consumed as articles of food, by the simple process of first treating them with benzoate of soda. It is to be remembered that the utilization of material which, without the use of benzoate of soda, must go to waste, represents a large margin of profit to certain selfishly commercialized interests, and this fact accounts in large part for the pressure brought to bear upon the government to reverse its original position.

Our government now allows the food manufacturers to use benzoate of soda for adulterating any of our foods and in this respect we are practically back where we were before the pure food law was passed.

This does not imply that the pure food and drug law is useless; far from that. If it did nothing but prevent misbranding of food and drugs, it would be valuable. We do not see mineral waters now advertised as curealls as in former days. Many of these waters claimed on their labels to cure all stomach, liver, kidney and bladder troubles, rheumatism, gout, and blood diseases. These claims the government declared false and fraudulent.

During these war times there is a fear that our Pure Food and Drugs Act will not be properly enforced. I am glad to note, however, that the Bureau of Chemistry of the Department of Agriculture is giving its full attention to the law, as some of its late reports clearly show. Efficient government protection along these lines, especially during the war, is of great benefit to the public, and is appreciated by the medical and pharmacal professions.

